# Evaluating the effectiveness of an exercise program based on the Adapted Utilitarian Judo program by analyzing fall competence in older adults

**DOI:** 10.1186/s12877-025-06058-6

**Published:** 2025-05-31

**Authors:** Marta Ortiz-Molina, Karin Strömqvist Bååthe, Óscar DelCastillo-Andrés, María del Carmen Campos-Mesa

**Affiliations:** 1 Department of Physical Education and Sport. Faculty of Education Sciences, Seville University, Seville, Spain; 2 School of Health and Welfare, Dalarna University, Falun, Sweden; 3 School of Health, Care and Welfare, Mälardalen University, Västerås, Sweden

**Keywords:** Self-efficacy, Falls, Injury prevention, Falling techniques, Well-being, Functional training, Adapted Utilitarian Judo

## Abstract

Population aging increases the risk of falls, particularly among postmenopausal women, which can negatively impact their quality of life. Research suggests that multifactorial exercise programmes can help reduce this risk. This study evaluates the effectiveness of an exercise program based on Adapted Utilitarian Judo (JUA) in enhancing older adults’ ability to safely manage falls; in this study called “fall competence”. Fall competence can be defined as having the knowledge how to fall safely, as well as possessing the motor skills and confidence (self-efficacy) to execute it effectively.

A quasi-experimental design was used, 22 participants, all female, in the Experimental Group (EG = mean age 75.77 ± 7.12 years) and 23 participants in the Control Group (CG = mean age of 75.96 ± 5.09 years). Self-efficacy and competence in backward (BF) and lateral falls (LF) were assessed pre- and post-intervention using the Strömqvist Bååthe Test. Sessions progressed from basic postures to falls from greater heights, all conducted under the guidance of a specialised instructor. Significant improvements in fall competence were observed in the experimental group (EG) after the 12-session intervention. Notable changes were found in backward falls (χ^2^(1) = 24.9, *p* = 0.001, V = 0.71) and lateral falls (χ^2^(1) = 28.6, *p* = 0.001, V = 0.80), with a large effect size, indicating a significant impact of the intervention on improving fall competence. In conclusion, this research brings to the scientific literature further evidence that judo-inspired exercise programs such as the JUA programme can be an effective tool to improve the quality of life of the older adult by contributing to improving their competences that allow them to remain independent in society.

**Supplementary Information:**

The online version contains supplementary material available at 10.1186/s12877-025-06058-6.

## Introduction

The World Health Organization estimates that the population over 60 will double between 2015 and 2050, heightening risks such as falls—a leading cause of trauma-related mortality [42]. Falls are influenced by individual and environmental factors [13], with women being more predisposed due to physiological and hormonal changes associated with menopause.

Alongside mortality, there is another factor directly related to the quality of life of older adults: frailty. Frailty further increases the risk of falls by compromising muscle strength, balance, and gait, contributing to greater dependency and sedentary behaviour [27, 33]. Multifactorial physical conditioning, with activities tailored to the individual [35], encompassing strength, endurance, balance, and flexibility, is recommended as an effective intervention against frailty [9, 14, 17].

In addition, together with the fragility derived from a fall, there is a no less serious psychological effect: the so-called fear of falling (FOF) (Tinetti, Richmond and Powell, [36]). For example, a study on postmenopausal women with osteoporosis highlights that fear of falling significantly affects their quality of life [32, 45]. Research has shown that contributing to improving the falls competence of older adults reduces FOF [6, 8].

In Spain, various programmes promote physical activity among older adults, such as the Activa programme, which aims to prevent chronic illnesses, or the programme *Exercise Takes Care of You*, which promotes health among those aged over 55 [21]. Another example is the programme *Moving is Caring For Yourself*, which emphasises the importance of exercise in improving physical condition and fostering socialisation [29]. Additionally, the recently introduced *Andalusian Physical Activity Prescription Plan* (PAPEF), aims to establish an effective system for prescribing physical activity and exercise, using digital tools to simplify health-based recommendations. The same programme also facilitates referrals for inactive individuals or those requiring specific exercise guidance to sports or community systems, ultimately improving their quality of life [20].

In this context, active ageing promotes health and social participation, addressing aspects such as community support and respect [38]. Supporting this idea of social inclusion, programs based on Judo are currently being developed, such as the *Judo Connecting Older and Younger Generations* (JOY) project, which aims to promote intergenerational judo practice as a tool to enhance the physical, social, and psychological well-being of older adults and young people, aligning with the previously mentioned concepts of support and respect within the community. Therefore, judo not only provides physical benefits through adapted techniques and exercises but can also strengthen the sense of community and belonging across generations. Participation in intergenerational programs enables older adults to regain lost social roles and enhance their well-being and life satisfaction [10]. These authors have demonstrated that, intergenerational judo has been shown to foster mutual learning, promoting values such as respect, discipline, and collaboration. Thus, the JOY project actively contributes to successful aging and the holistic development of participants, establishing itself as an innovative intervention model in the fields of sports and health.

However, despite the availability of exercise programmes, the necessary development of skills such as falling techniques, which are crucial for injury prevention, are often overlooked. Disciplines such as yoga and Tai Chi have been shown to be effective in enhancing balance, strength, and flexibility, thereby reducing the risk of falls among older adults [22, 34, 40, 44]. However, these practices do not specifically train individuals in techniques to protect themselves from injury in the event of a fall.

Recent research, however, highlights the usefulness of fall prevention workshops and judo-based programmes to teach safe falling techniques, empowering older individuals [6, 37].

In addition to the physical benefits, martial arts and combat sports have been widely studied for their positive impact on mental health. Judo has shown not only to improve physical fitness but also to contribute to the development of self-efficacy, self-esteem, and emotional resilience [39]. Its emphasis on self-control and discipline supports stress and anxiety reduction [26], while fostering self-confidence through the mastery of physical and technical challenges [11]. Additionally, as a controlled-contact sport, judo enhances emotional regulation and the perception of personal competence, which are key factors in promoting psychological well-being in adult and older populations [23]. Recent studies have highlighted the potential of judo as a therapeutic tool for developing self-related psychological skills, particularly in active aging programs [6].

Da Silva Musa & Pombo Menezes [12], explore the impact of coaching practices on sports performance as well as athletes’ psychological and pedagogical development. They underscore the importance of continuous training and knowledge exchange among coaches and researchers, advocating collaborative spaces to enhance practices and optimise training quality. These proposals have been supported by the International Consensus on Safe Falls for Older People through Judo, held in November 2024 in Japan [5].

Findings from this consensus statement supports the implementation of programmes, such as Adapted Utilitarian Judo (JUA) in Spain, which focuses on daily and functional movement while enhancing falling competence in older adults [9], and *Judo4Balance* in Sweden, which targets strength and balance improvements to prevent falls as well as teaching safe falling and landing techniques to prevent injuries from unintentional falls [2, 6, 16, 25]. Other programmes include *Dynamic Balance for Life* in Australia, which in addition to balance exercises teaches how to get up after a fall [18], and *Yawara-chan Taiso* in Japan, designed to prevent falls in older adults [30]. In Quebec, judo courses have improved physical fitness and reduced the fear of falling (Latest Canada, [24]). Similar initiatives have been adapted in the UK (British [4]), Azerbaijan [5], and Japan, through the *Judo Kenko Taiso* project, which aims to enhance stability and cognitive function in older individuals [43].

The Japanese Judo Federation runs a risk assessment and judo-based physical training programme to improve fitness and safe falling techniques. It also investigates the use of specific judo exercises to enhance strength, balance, and coordination among older adults [5]. Meanwhile, Judo Flanders offers home sessions to teach falling techniques, as detailed on its website (Judo Flanders, n.d. [19]), and the *Educating Judo Coaches for Older Practitioners (*EdJCO*)* project focuses on training coaches to work with this demographic [11]. Additionally, the *Nijmegen Fall Prevention Programme* in the Netherlands incorporates *ukemi* techniques, demonstrating effectiveness in reducing fall risk and boosting mobility confidence in older adults [41].

For this study, we selected the JUA program due to its scientific foundation, methodological structure, and proven effectiveness in injury prevention and autonomy enhancement in older adults [9]. It employs a progressive methodology that adapts exercises based on factors such as center-of-gravity height, rotation axes, number of participants, and execution speed. Its applicability to daily life makes it an effective option for teaching falling safely without injury. Through this approach, Adapted Utilitarian Judo (JUA) was chosen for the intervention in older individuals because of its specific focus on teaching safe falling techniques, which unlike other sports that enhance balance and strength without directly addressing falling competence. Thus, previous research has shown that judo-based programmes, such as Judo4Balance (Sweden) and Yawara-chan Taiso (Japan), enhance older adults’ safety by improving their fall-related competencies.

This study introduces key innovations in falls prevention for older adults because of its proactive approach. Methodologically, it uses Judo Utility Adapted Judo (JUA), which incorporates judo techniques to teach participants how to fall safely, differentiating it from other prevention-only approaches by focusing on balance and strength work [7, 17, 35]. In addition, it establishes a three-level progression tailored to participants’ abilities, emphasising impact management during a fall, an aspect less explored in the literature [31]. Furthermore, it employs the Strömqvist-Bååthe Falling Competence Test, a specific tool for assessing fall competence, addressing a gap in previous studies that relied on general mobility tests such as the Timed Up and Go [28]. Moreover, it proposes integrating JUA into physical exercise prescription programs, a strategy not previously explored.

The primary aim of this article is to assess whether a sample of older adults, following a multifactorial fall prevention exercise program based on the JUA programme, can develop the motor skills needed to mitigate injuries and optimise physical and psychological responses to falls.

Hypothesis 1: Participation in a fall prevention exercise program based on the Adapted Utilitarian Judo (JUA) program will significantly improve older adults’ competence in backward falling techniques.

Hypothesis 2: Participation in a fall prevention exercise program based on the Adapted Utilitarian Judo (JUA) program will significantly improve older adults’ competence in lateral falling techniques.

Hypothesis 3: Participation in a fall prevention exercise program based on the Adapted Utilitarian Judo (JUA) program will enhance self-efficacy in older adults.

## Design and methodology

### Study design

The study follows a quasi-experimental design, incorporating measurements taken before and after the intervention in both the Control Group (CG) and Experimental Group (EG). The sample was selected through non-probabilistic, incidental sampling, relying on convenience for accessibility.

### Participants

The study included 45 participants, all female, with 22 in the Experimental Group (EG), aged from 63 to 90 years (average age 75.77 ± 7.12 years). Anthropometric data for the EG indicated a body mass index (BMI) range of 26.60 to 44.55 kg/m^2^ (average BMI of 35.07 ± 4.71 kg/m^2^) and muscle mass ranging from 15.3% to 33.1% (mean muscle mass of 22.23 ± 4.17%). The Control Group (CG) had 23 participants, aged between 61 and 83 years (mean age of 75.96 ± 5.09 years). The CG’s BMI ranged from 26.24 to 43.37 kg/m^2^ (mean BMI of 33 ± 3.98 kg/m^2^), with muscle mass percentages from 16.1% to 29.1% (mean muscle mass of 21.14 ± 3.82%).

Regarding profession, in the EG, 21.7% of the participants were housewives, and 8.7% were retired teachers and seamstresses. The remaining percentage is distributed among various other professions, including commercial workers, cleaners, and nurses. Similarly, in the CG, 58.3% of the subjects were housewives, with other occupations such as teachers and cleaners also being represented.

In terms of marital status, 26.1% of the participants in the EG were married and living with a partner, while 17.4% were widowed and living alone. In the CG, 20.8% were widowed and living alone, and 12.5% were widowed but living with a companion. It is crucial to highlight that widowed individuals living alone have a considerably higher risk of adverse consequences in the event of falls compared to those who, despite being widowed, live with a companion.

### Inclusion/exclusion criteria

The inclusion criteria required participants to be 60 years or older, of both sexes, without pre-existing pathologies, and without diagnosed diseases that would prevent them from exercising. Those excluded were individuals who, for medical reasons, were advised against physical exercise, those with congestive heart failure, chest pain, dizziness, or angina during exercise, or uncontrolled hypertension (160/100 mmHg).

### Instruments

The intervention took place in a pavilion at the sports center of the Municipal Sports Institute of Tiro de Línea in Seville (Spain), with tests conducted in an adjacent room equipped for anthropometric measurements and physical fitness evaluations. Measurements included height, weight, and body mass index (BMI), using a measuring tape and a scale with a body analyzer. The session began with anthropometric measurements and personal questions, followed by tests using the corresponding instruments.

**Fig. 1:**
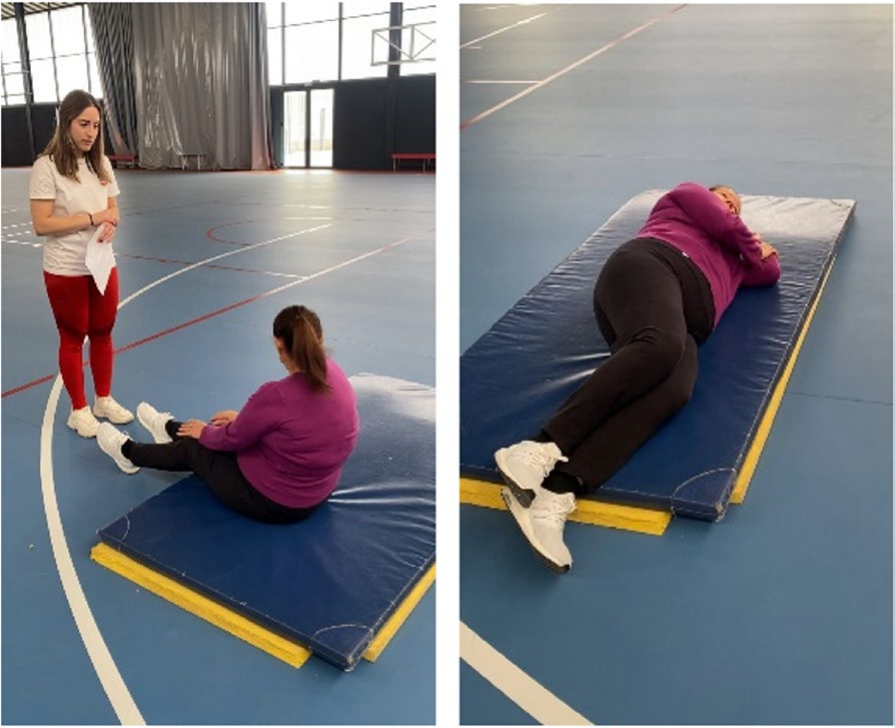
Evaluation of the Lateral Fall measurement test (SBFC). Source: Own elaboration

- Strömqvist Bååthe Falling Competence (SBFC) Test [1]:

The Strömqvist Bååthe Test is a tool designed to measure fall competence. It assesses self-efficacy, motor skills, and fall technique, defining fall competence as the ability to theoretically understand how to fall, the confidence to execute a fall, and the skills necessary to perform a fall safely (Arkkukangas et al., [1]). The test is divided into three sections to evaluate backward, lateral, and forward falls. However, this dissertation will focus solely on the assessment of backward and lateral falls (Fig. 1).

For self-efficacy, participants are asked the following question: "How confident are you...?" The question's ending depends on the type of fall being evaluated. If the participant responds positively, they proceed with the test; if they respond negatively, the test is discontinued. This process applies to both backward (BF) and lateral falls (LF).

Regarding motor skills and fall technique, once a participant demonstrates self-efficacy by responding positively, the test is conducted. If the participant decides not to complete a section of the test, they are not required to continue.

The technical procedure for evaluating motor skills and falling technique begins with assessing the backward fall. Participants are first asked to lie down on their back on a mat, lift their head, and then attempt to get up to standing again independently. If successful, they earn 1 point. If unsuccessful, the procedure is halted, and the specific failure (such as inability to lift the head, failure to lay down or stand up independently) is documented in the test protocol.

Next, participants proceed to sit down and fall backward. Successfully completing this task earns them 2 points. In the subsequent stage, participants perform a safe backward fall from a squatting position, earning 3 points if executed correctly. The final stage involves performing a safe backward fall from a standing position, which is worth 4 points.

For each position (in stages 2 to 4), participants are assessed based on a checklist of 5-6 critical manoeuvres that could potentially lead to harm at the next stage. These include failure to hold up the head, bracing the fall with an arm or elbow, not rolling, or any other harmful action. Each harmful manoeuvre is noted and considered during the scoring process; and just one mistake will lead to failing that part of the test and the test leader will stop the test at this point.

The test methodology prioritises safety and a logical progression in the difficulty and height of the falls. The test is immediately stopped at any sign of risk or unsafe manoeuvres, and the supervising teste leader must be prepared to halt the test and provide assistance if needed. 

### Intervention

The study was conducted from March to May 2024, over 12 sessions. All participants received comprehensive information regarding the intervention, including its design, procedures, duration, and potential benefits. Written informed consent, image release, and health declaration forms were obtained prior to participation. This intervention lasted for 4 weeks, with 3 sessions per week, and each session lasted 60 min, divided into three parts. The first part consisted of a 10-min warm-up, which included joint mobility exercises, dynamic stretching, fine motor skills (putting on the judo belt), and the initial ritual (standing judo greeting). The main part (40 min) focused on multicomponent exercises distributed over the 12 sessions. These blocks progressed from practicing basic fall techniques and strength exercises to refining advanced fall techniques and training to improve balance, coordination, and muscular stability. The final part (10 min) consisted of a cool-down and static stretching to relax the muscles, improve flexibility, and promote recovery.

The meticulously designed 12-session workshop was structured into three progressive phases. Initially, participants were introduced to the basic concepts of judo, from the greeting to fundamental postures and gripping techniques, while working on muscle strengthening and balance. In the intermediate sessions, fall techniques were further developed, and strength exercises were intensified, with a focus on coordination and balance. Finally, in the later stages, more complex fall techniques, such as falls from a standing position, were introduced, while continuing to develop strength and motor skills through more challenging exercises. This structured approach allowed for progressive improvement in physical performance and competence in fall techniques.

The intervention was conducted by a student undertaking this master’s thesis, a highly experienced judo instructor (1st DAN black belt), who carefully structured the sessions according to the level and individual needs of the participants. The approach was progressive, using partner exercises to foster motivation and support among participants.

### Data analysis

Data analysis was performed using SPSS version 29 statistical software. A descriptive statistical analysis was performed to determine the characteristics of the sample. In addition, considering that the sample is smaller than 50 and that the normality test to assess the distribution of the data yielded a *p*-value < 0.05, which indicated that the data did not follow a normal distribution, non-parametric tests were applied. Specifically, the Wilcoxon signed-rank test for related samples was used to compare pre-post in the EG and the Mann–Whitney U test for independent samples EG and CG. Effect sizes and degrees of freedom are indicated [15].

## Results

### Analysis of fall competence (Strömqvist Bååthe Test)

#### Self-efficacy in fall competence

The results show (Table 1) significant differences in several comparisons, highlighting changes in self-efficacy between the CG and EG in the pre- and post-intervention phases. Regarding self-efficacy in performing a fall from a squatting position (3aBF), a significant change was observed (χ2(1) = 17.8, *p* = 0.001) with a large effect (V = 0.63), indicating that the experimental group (EG) exhibited a notable increase in self-efficacy after the intervention. A similar pattern was found in self-efficacy for performing a fall from a standing position (4aLF) (χ2(1) = 19.1, *p* = 0.001, V = 0.65) and in subjects’ self-efficacy for lateral falling from a squatting position (3aLF) (χ2(1) = 23.5, *p* = 0.001, V = 0.72), both with large effects, suggesting that the intervention had a substantial impact.

**Table 1:** Differences between CG and EG in SBFC Test in pre and post phase regarding self—efficacy

		CG (*n* = 23)				EG (*n* = 22)				χ^2^(df)	*p*-value	V
		No/Unsure		Yes		No/Unsure		Yes				
		*N*	%	*N*	%	*N*	%	*N*	%			
1aBF	Pre	10	43.5	13	56.5	2	9.1	20	90.9	6.80(1)	0.009	0.39
	Post	12	52.2	11	47.8	5	22.7	17	77.3	4.15(1)	0.042	0.30
2aBF	Pre	14	60.9	9	39.1	9	40.9	13	59.1	1.79(1)	0.181	0.20
	Post	14	60.9	9	39.1	5	22.7	17	77.3	6.71(1)	0.010	0.39
3aBF	Pre	21	91.3	2	8.7	20	90.9	2	9.1	0.002(1)	0.963	0.01
	Post	22	95.7	1	4.3	8	36.4	14	63.6	17.8(1)	0.001	0.63
4aBF	Pre	22	95.7	1	4.3	22	100.0	-	-	0.98(1)	0.323	0.15
	Post	23	100.0	-	-	9	40.9	13	59.1	19.1(1)	0.001	0.65
1aLF	Pre	12	52.2	11	47.8	4	19.0	17	81.0	5.21(1)	0.023	0.34
	Post	13	56.5	10	43.5	5	22.7	17	77.3	5.35(1)	0.021	0.35
2aLF	Pre	16	69.6	7	30.4	10	45.5	12	54.5	2.68(1)	0.102	0.24
	Post	15	65.2	8	34.8	5	22.7	17	77.3	8.22(1)	0.004	0.43
3aLF	Pre	22	95.7	1	4.3	20	90.9	2	9.1	0.41(1)	0.524	0.10
	Post	23	100.0	-	-	7	31.8	15	68.2	23.5(1)	0.001	0.72
4aLF	Pre	22	95.7	1	4.3	22	100.0	-	-	0.98(1)	0.323	0.15
	Post	23	100.0	-	-	9	40.9	13	59.1	19.1(1)	0.001	0.65

*V* Cramer’s V, *low effect* 0.10, *medium effect* 0.30, *large effect* 0.50

1aBF = How confident are you laying down on your back and lift your head from the mat (tuck chin in) and put your arms beside you (palms down) and then stand up again?

2aBF = How confident are you sitting down on your buttocks (legs forward) and fall backward

3aBF = How confident are you falling backwards from a squatting position?

4aBF = How confident are you falling backwards from a standing position?

1aLF = How confident are you laying down on your side, lift your head and roll to the side?

2aLF = How confident are you sitting down on your buttocks (legs forward) and fall sideways?

3aLF = How confident are you falling sideways from a squatting or one kneeling position?

4aLF = How confident are you falling sideways from a standing up position?

Additionally, for lateral fall 1aLF, which assesses whether participants feel confident lying on their back, lifting their head off the mat, placing their arms to the sides, and standing up unassisted (χ2(1) = 5.35, *p* = 0.021, V = 0.35), as well as for lateral fall 2aLF, which assesses confidence in falling backward after sitting (with legs extended forward) without specific instructions (χ2(1) = 8.22, *p* = 0.004, V = 0.43), moderate-to-large effects were observed, reinforcing the trend of improvement in the experimental group.

In contrast, some values did not reach significance, such as self-efficacy for falling backward after sitting (2aBF) (χ2(1) = 1.79, *p* = 0.181, V = 0.20), indicating a smaller difference between groups in this condition. Overall, the data suggest that the intervention had a positive impact, particularly in key aspects of self-efficacy.

#### Backward fall and lateral fall competence

In relation to Table 2 the results indicate significant differences in several comparisons, highlighting changes in fall competence between the CG and EG groups in the pre- and post-intervention phases. In falling backward after sitting (legs forward) without instructions (2bBF) post, a significant change was observed (χ2(1) = 24.9, *p* = 0.001) with a large effect (V = 0.71), suggesting that the experimental group (EG) experienced a notable improvement in fall competence following the intervention. Similarly, substantial changes were found in falling backward after squatting without instructions (3bBF) post (χ2(1) = 21.2, *p* = 0.001, V = 0.69) and lateral falling from a squatting or kneeling position without instructions (3bLF) post (χ2(1) = 28.6, *p* = 0.001, V = 0.80), both with large effect sizes, reinforcing the positive impact of the intervention.

**Table 2:** Pre and Post results of the EG and CG in the SBFC test on Falling Competence

		CG				EG				χ^2^(df)	*p*-value	V
		No		Yes		No		Yes				
		*N*	%	*N*	%	*N*	%	*N*	%			
1bBF	Pre	10	43,5%	13	56,5%	7	31,8%	15	68,2%	0,65(1)	0,42	0,12
	Post	14	60,9%	9	39,1%	5	22,7%	17	77,3%	6,71(1)	0,01	0,39
2bBF	Pre	17	73,9%	6	26,1%	17	77,3%	5	22,7%	0,07(1)	0,793	0,04
	Post	22	95,7%	1	4,3%	5	22,7%	17	77,3%	24,9(1)	0,001	0,71
3bBF	Pre	21	91,3%	2	8,7%	22	100,0%	-	-	2,00(1)	0,157	0,21
	Post	23	100,0%	-	-	8	36,4%	14	63,6%	21,2(1)	0,001	0,69
4bBF	Pre	22	95,7%	1	4,3%	22	100,0%	-	-	0,98(1)	0,323	0,15
	Post	23	100,0%	-	-	12	54,5%	10	45,5%	13,4(1)	0,001	0,55
1bLF	Pre	13	56,5%	10	43,5%	7	31,8%	15	68,2%	2,78(1)	0,095	0,25
	Post	15	65,2%	8	34,8%	5	22,7%	17	77,3%	8,22(1)	0,004	0,43
2bLF	Pre	19	82,6%	4	17,4%	15	68,2%	7	31,8%	1,27(1)	0,26	0,17
	Post	23	100,0%	-	-	5	22,7%	17	77,3%	28,6(1)	0,001	0,80
3bLF	Pre	22	95,7%	1	4,3%	21	95,5%	1	4,5%	0,001(1)	0,974	0,00
	Post	23	100,0%	-	-	8	36,4%	14	63,6%	21,2(1)	0,001	0,69
4bLF	Pre	22	95,7%	1	4,3%	22	100,0%	-	-	0,98(1)	0,323	0,15
	Post	23	100,0%	-	-	12	54,5%	10	45,5%	13,4(1)	0,001	0,55

*V* Cramer’s V, *low effect* 0.10, *medium effect* 0.30, *large effect* 0.50

1bBF = Lies down on the back, lifts head off the mat, places the arms at the side, and then stands up again without assistance

2bBF = Backward Fall from sitting with legs forward

3bBF = Backward Fall from squatting

4bBF = Backward Fall from standing

1bLF = Lies down on the side, can lift the head and roll back and forth to the side

2bLF = Lateral Fall from sitting with legs forward

3bLF Lateral Fall from squatting or sitting on the knees

4bLF = Lateral Fall from standing up

Additionally, falling backward from standing without instructions (4bBF) post (χ2(1) = 13.4, *p* = 0.001, V = 0.55) and rising and falling on the side without instructions (4bLF) post (χ2(1) = 13.4, *p* = 0.001, V = 0.55) also show significant differences with large effects, indicating that the intervention meaningfully contributed to improving participants’ ability to execute falls safely. Moreover, lying on the back, lifting the head, placing the arms at the sides, and lifting off the ground without assistance (1bBF) post (χ2(1) = 6.71, *p* = 0.01, V = 0.39) and falling on the side from a sitting position (legs forward) without instructions (2bLF) post (χ2(1) = 8.22, *p* = 0.004, V = 0.43) presented moderate-to-large effects, suggesting consistent improvements in fall competence in the experimental group.

In contrast, some comparisons did not reach statistical significance, such as falling backward after sitting (legs forward) without instructions (2bBF) (χ2(1) = 0.07, *p* = 0.793, V = 0.04), indicating minor differences between groups in the pre-intervention phase. Overall, these findings suggest that the intervention had a substantial positive effect on fall competence, particularly in key performance aspects.

The results in Table 3 show that, in the pre-intervention phase, no significant differences were found between the control group (CG) and the experimental group (EG) in competence and the LF technique (U = 241, *p* = 0.781) and in competence and the BF (U = 187, *p* = 0.115), indicating that both groups had similar performance before the intervention.

**Table 3:** Results of the total score competence in the SBFC-Test Pre and Post

	CG		EG		U-MW	*p*-value	rbis
	M	SD	M	SD			
Competence and LF PRE	0.96	1.1	0.91	0.8	241	0.781	0.05
Competence and LF POST	0.48	0.6	2.64	1.6	84	0.001	0.67
Competence and BF PRE	0.70	1.0	1.05	0.9	187.5	0.115	0.26
Competence and BF POST	0.39	0.5	2.64	1.6	80	0.001	0.68

*U-MW* U Mann–Whitney, *rbis* rank biseral correlation, *low effect* 0.10, *medium effect* 0.30, *large effect* 0.50

However, in the post-intervention phase, significant differences were observed for both falling competence. For competence and LF, the experimental group showed a marked improvement (M = 2.64,SD = 1.6) compared to the control group (M = 0.48,SD = 0.6), with a significant difference (U = 84, *p* = 0.001). Similarly, in competence and BF, the experimental group also outperformed the control group in the post phase (U = 80, *p* = 0.001), reflecting a substantial improvement after the intervention.

The effect size (rbis), as we can see after the intervention is large. This suggests that the intervention had a relevant impact on the improvement of falling competence in the experimental group.

As can be seen in Table 4 both types of falls (LF and BF) show significant differences between PRE and POST measurements, with a large effect size (rbis > 0.9) for both. The lateral fall seems to have a slightly larger mean difference (2.50 vs. 2.00) than the backward fall, although both techniques show significant improvements in competence according to the Wilcoxon test results.

**Table 4:** Paired differences in EG for LF and BF

	W-R	*P*-value	MD	SD	r_bis_
Competence and LF PRE-POST	151	<.001	2.50	0.3	0.974
Competence and BF PRE-POST	152	<.001	2.00	0.3	0.980
*W-R* Willconson rank, *MD *Mean difference, r_*bis*_rank biseral correlation, *low effect*: 0.10, *Medium effect*: 0.30, *Large effect*: 0.50					

## Discussion

After analysing the results, the main finding was that participants who attended the fall-training exercise session over 12 sessions demonstrated significant improvements compared to the control group (CG). The participants in the experimental group (EG) showed increased levels of self-efficacy, enabling them to feel confident to try and subsequently; successfully perform all types of falls with proper skill and technique, both the backward fall (BF) and the lateral fall (LF). At least about half of the participants successfully completed the tasks. These data are consistent with the findings of Tinetti, Richman, and Powell [36], Campos-Mesa et al. [8], and Callan et al. [6], who report that judo-based programmes can enhance self-efficacy in older adults.

By improving psychological factors that are crucial for older adults, such as self-efficacy, judo-based interventions can play a key role in promoting autonomy and quality of life, particularly in fall prevention. According to Bandura [3], self-efficacy influences motivation and the ability to face physical challenges. In the context of aging, studies by Tinetti, Richman, and Powell [36] and Campos-Mesa et al. [8] have demonstrated that higher self-efficacy in movement execution reduces fear of falling (FOF) and enhances fall competence. Interventions based on multifactorial programs and adapted judo have proven effective in developing this motor confidence [6].

In our study, the EG’s fall competence showed significant improvement in post-intervention assessments. These findings are consistent with those of Arkkukangas et al. [1] and Vertongen et al. [39], who also reported significant improvements in fall competence following a similar intervention. In the study by Arkkukangas et al. [1], positive outcomes were observed following a 10-week intervention based on the Judo4Balance programme for both BF and LF, aligning with the findings of our research.

It is important to highlight the significant improvements observed in the EG compared to the CG, which supports the effectiveness of teaching fall competence by using judo-based exercise programs which have a long tradition of teaching effective falling techniques. These results are in line with those reported by Arkkukangas et al. [2], who also identified significant improvements in fall competence after a Judo4Balance exercise intervention. Their study included a local judo club, a health centre, and a group of employed older adults. While the judo club showed the most positive effects due to its favourable environment for teaching proper falling techniques, all three groups experienced significant improvements in fall competence with the same intervention plan, similar to the outcomes of this study.

In a meta study by Sosnoff & Moon (2017) it is shown that most landing techniques can reduce impacts from falls in all directions. This study is in alignment with our study, emphasizing the importance of enhancing both backward and lateral fall competence.

Learning falling techniques, such as backward or lateral falling, has been shown to reduce hip impact in older individuals, which can significantly decrease the likelihood of a fracture occurring [16]. A hip fracture in an older adult resulting from a fall possesses a high risk of permanently reducing the mobility and thus putting the individual at increased risk of being less active and risking increased social isolation. According to the WHO, individuals *who fall and suffer a disability, particularly older people, are at a major risk for subsequent long-term care and institutionalization* [42].

This study’s proposition—that integrating falling techniques into exercise programmes for older adults may contribute significantly to long-term quality of life—is supported by previous research [5, 16, 25, 43], which highlights benefits such as reduced fear of falling, increased confidence, and a lower risk of injury in the event of an unintentional fall.

The main limitations of this study include the small sample size and all participants being female, the quasi-experimental design that may introduce biases and limit the generalisability of the results, and the lack of long-term follow-up to assess the sustainability of the programme’s effects. We are aware of this and will try to continue to contribute to scientific progress with further interventions.

## Conclusion

In conclusion, we can confirm our three hypotheses that the fall-training exercise program based on the JUA, implemented over 12 sessions with a group of older adults, was effective in improving both backward and lateral falls competence. Furthermore, participants demonstrated a significant increase in self-efficacy, which translated into successful execution of both backward and lateral falls. Analysing the Strömqvist Bååthe Falling Competence Test from the first self-efficacy half-step perspective and connecting it to successful execution is an important addition to the literature in this emerging field of studying falling competence. Moreover, notable improvements were also observed in the subsequent step of performing the falling technique including the safe landing. Fall competence showed considerable improvement in the assessments conducted after the intervention, supporting the efficacy of the intervention.

Therefore, the integration of judo-based exercise programmes (such as JUA) including the teaching of safe falling and landing techniques complementing already proven effective sport activities for older adults, such as maintenance fitness, pilates and similar exercises, could be an effective strategy to improve older adults’ self-confidence and motor skills to fall safely.

Adding a new dimension to the SBFC test by analyzing the pre-step of falling self-efficacy—asking participants if they felt comfortable performing the fall—provides valuable insights into how fall training can enhance self-confidence and encourage further practice. Future studies should further validate the Strömqvist Bååthe Falling Competence Test, particularly the “half-step” question, as this study shows a significant increase in confidence among participants in the exercise group compared to the control group.

This study contributes to the scientific literature by providing additional evidence that judo-based exercise programs, such as the JUA program, can serve as a valuable tool for enhancing the quality of life for older adults. Specifically, these programs contribute to improved motor self-confidence (self-efficacy) and falling competence, that can be an important resource for older individuals to remain independent and physically empowered.

## Supplementary Information

Supplementary Material 1.

## Data Availability

Data is provided within the manuscript and any additional anonymized sequence data that support the findings of this study is safely deposited at University of Seville and can be provided upon request.

## Abbreviations

BF: Backward fall
CG: Control Group
EG: Experimental Group
JOY: Judo connecting older and younger generations
JUA: Adapted Utilitarian Judo
LF: Lateral fall
PAPEF: Andalusian Physical Activity Prescription Plan
SBFC Test: Strömqvist Bååthe Fall Competence Test
WHO: World Health Organisation
