# Supplementary material for: Evaluating the effectiveness of an exercise program based on the Adapted Utilitarian Judo program by analyzing fall competence in older adults
Source: BMC Geriatr. 2025 May 31;25:395. doi: 10.1186/s12877-025-06058-6 (PMC12125915; doi:10.1186/s12877-025-06058-6)
Supplement: Supplementary file 1 — Supplementary Material 1. [file 12877_2025_6058_MOESM1_ESM.pdf]

“Strömqvist-Bååthe Test” –
Measuring Falling Competence (SBFC – Test)

DALARNA
UNIVERSITY

Background: The interest in “break fall” training (inspired from martial arts training such as Judo, Ju-Jitsu and Aikido) as a method of mitigating injuries from unintentional falls is increasing among the scientific community. This test was developed based on the need for a simple standardized test that can be used by both researchers and the trainers/coaches to evaluate the effects of different Fall Preventive Exercise programs where falling techniques are included. The test has undergone ethical approval (from the Swedish Ethical Review Authority) and has been used in several scientific studies evaluating the learning of falling techniques by over 450 working age adults and senior citizens but could also be used as a method to evaluate the falling literacy of children.

Who can use the test? As of our understanding, this is the first publicly available test free to use for the scientific community, as long as the correct name of the test is cited and a simple registration by e-mail is made to the point of contacts: stating when and how the test will be carried out. Contact details of at least one researcher/coach should also be provided. (training on how to use the test can be made upon request in a short session approximately 30 min through zoom)

Point of contact: Karin Strömqvist Bååthe, Dalarna University, Falun Sweden, e-mail: ksb@du.se (phone: +46 730-89 61 62) or professor Michail Tonkonogi, Dalarna University, e-mail: mtn@du.se

What does the test measure? The test measures both the falling technique of the test participant in combination with the self-efficacy and motor skill. “Fall Competence” in the context of this test can be described as: ***“the combination of knowing intellectually how to fall, having the confidence to perform the fall as well as having the motor skill and strength to perform the fall in a safe manner according to the martial arts falling and landing strategies”***.

Which falls can be tested? The test is developed in three sections to assess backwards, sideways and forwards falling competence independently. The test can be used for assessing only one, two or all three falling directions based on assessment need. Please observe that there are two approved forward falling strategies according to this test. The rolling and non-rolling forward break fall. Concerning falling styles the traditional judo break-fall with arm/s falling alongside the body in an approximate 45% angle slapping the mat as well as the modified break fall used in some studies not using the arms for slapping the mat or slapping very softly has been accepted for the falls. However, the authors of these test have not seen any negative consequences of teaching the “classic” judo falls to healthy or pre-frail older beginners although it requires some more coordination. On the contrary, slapping the arms help the persons keep the head up, mitigate straight arm or elbow “bracing reflexes” and balances the roll. However, more research is needed in this field.

Equipment Needed: The test is performed on a soft martial arts puzzle mat (normally between 3 – 6 cm thick) or on a tatami in a martial arts school (dojo). The test subject should preferably be barefoot or having non-slippery socks as well as wear soft and flexible clothing (for example: gi or exercise clothes)

Test Methodology and Safety: The test follows a logical progression of increased difficulty and falling height, with participant’s trust and “safety first” as a key ingredient. The test should always be discontinued as soon as the test participant does not manage to perform the fall exercise or shows any sign of hesitation or worry to perform the next step. Do NOT as a test leader push the participant to “try” the falling at a level/height where the participant does not feel comfortable to try; and always stop the test if the participant preforms the test and you identify any type of harmful maneuvers. As soon as the test leader identifies a potentially harmful maneuver the test is stopped. The test leader needs to be prepared to catch the individual as well as help the test subject to rise from the floor if needed.

Name/Code: __________________ Date: _______ Testleader: _____________________ Place: ________________

“Strömqvist - Bååthe Test” - Measuring Falling Competence - Backwards:

1a) How confident are you laying down on your back and lift your head from the mat (tuck chin in) and put your arms beside you (palms down) and then stand up again?

☐ YES, SURE (go to 1b) ☐ UNSURE (stop test) ☐ NO/REFUSES (stop test)

1b) Test-participant does the exercise – Lays down on the back, lifts the head of the mat and lays arms beside and then stands up again from the floor without help.

☐ Successful (go to 2a) ☐ Cannot lift the head off the mat (stop test)
☐ Cannot get down on the mat (stop test) ☐ Cannot get up from the mat independently (stop test)

2a) How confident are you sitting down on your buttocks (legs forward) and fall backward?

☐ YES, SURE (go to 2b) ☐ UNSURE (stop test) ☐ NO/REFUSES (stop test)

2b) Performs the exercise – Falls backward from sitting (legs forward) no instruction given:

☐ Successful (go to 3a) ☐ Not rolling up with legs (stop test) ☐ Puts down hand/hands (stop test)
☐ Puts down elbow/lower arm/s (stop test) ☐ Does not hold up the head (stop test)
☐ Other injury prone maneuver: ____________________________________(stop test)

3a) How confident are you falling backwards from a squatting position?

☐ YES, SURE (go to 3b) ☐ UNSURE (stop test) ☐ NO/REFUSES (stop test)

3b) Performs the exercise – Falls backward from squatting (no instruction given)

☐ Successful (go to 4a) ☐ Not rolling up with legs (stop test)
☐ Puts down hand/hands (stop test)
☐ Puts down elbow/lower arm/s (stop test) ☐ Not holding up head (stop test)
☐ Other maneuver which could be harmful: ____________________________________(stop test)

4a) How confident are you falling backwards from a squatting position?

☐ YES, SURE (go to 4b) ☐ UNSURE (stop test) ☐ NO/REFUSES (stop test)

4b) Performs the exercise – Falls backward from standing (no instruction given)

☐ Successful (=4p) ☐ Does not bend knees (stop test) ☐ Not rolling up with legs (stop test)
☐ Puts down hand/hands (stop test) ☐ Puts down elbow/lower arm (stop test)
☐ Does not hold up head (stop test) ☐ ”Crawling” instead of rolling backwards (stop test)
☐ Other injury prone maneuver: (describe) ____________________________________(stop test)

POINTS BACKWARD FALL ______________

Scale:
0 point = Fails exercise 1a = Refuses, unsure or cannot lay down on the floor and rise independently or fails 1b.

1 point = Successful Exercise 1b = Can successfully lay down on the floor and rise independently but fails 2a or 2b.

2 points =Successful Exercise 2b = Can successfully fall backwards from sitting down on the floor but fails 3a or 3b

3 points = Successful Exercise 3b = Can successfully fall backwards from a squatting position but fails 4a or 4b.

4 points = Can successfully fall backwards from a standing position without any harmful maneuver (exercise 4b).

“Strömqvist-Bååthe Test” for measuring Falling Competence Backwards, Sideways & Forward: Copyright: K. Strömqvist Bååthe & M. Tonkonogi, University of Dalarna, developed 2018 - 2019. English translation May 2023.
Tests may be used free of charge by trained test leaders with the acknowledgement of the correct test name as well as simple registration of the use by e-mail to: ksb@du.se stating: when and how the test will be used as well as name of point of contact, e-mail and name of institution/club/company

Name/Code: ________________ Date: ______ Testleader: ____________________ Place: ______________

"Strömqvist - Bååthe Test" - measuring Falling Competence - Sideways:

1a) How confident are you laying down on your side, lift your head and roll from side to side?

☐ YES, SURE (go to 1b) ☐ UNSURE (stop test) ☐ NO/REFUSES (stop test)

1b) Performs the exercise – Lays down on the side, can lift head and roll from side to side.

☐ Successful (go to 2a) ☐ Cannot get down on the mat (stop test)
☐ Cannot lift the head off the mat (stop test) ☐ Cannot roll from side to side (stop test)
☐ Cannot get up from the mat independently (stop test)

2a) How confident are you sitting down on your buttocks (legs forward) and fall sideways?

☐ YES, SURE (go to 2b) ☐ UNSURE (stop test) ☐ NO/REFUSES (stop test)

2b) Performs the exercise – Falls sideways from sitting down (legs forward) no instruction given:

☐ Successful (go to 3a) ☐ Puts down hand/hands (stop test)
☐ Does not hold up head (stop test) ☐ Falls on stomach (stop test)
☐ Hits the shoulder (stop test) ☐ Falls flat on the back (stop test)
☐ Other injury prone maneuver: (describe) ______________________________(stop test)

3a) How confident are you falling sideways from a squatting or one kneeling position (one knee up)?

☐ YES, SURE (go to 3b) ☐ UNSURE (stop test) ☐ NO/REFUSES (stop test)

3b) Falls sideways from squatting or kneeling position, no instruction given:

☐ Successful (go to 4a) ☐ Puts down hand/hands (stop test)
☐ Does not hold up the head (stop test) ☐ Falls on the stomach (stop test)
☐ Hits the shoulder (stop test) ☐ Falls flat on the back (stop test)
☐ Other injury prone maneuver: (describe) ______________________________(stop test)

4a) How confident are you falling sideways from a standing up position?

☐ YES, SURE (go to 4b) ☐ UNSURE (stop test) ☐ NO/REFUSES (stop test)

4b) Stands up and falls sideways, no instruction is given:

☐ Successful (=4p) ☐ Puts down hand/hands (stop test) ☐ Does not hold up the head (stop test)
☐ Falls on the stomach (stop test) ☐ Hits the shoulder (stop test)
☐ Falls flat on the back (stop test) ☐ Does not bend leg/s and lower center of gravity (stop test)
☐ Other injury prone maneuver: (describe) ______________________________(stop test)

POINTS BACKWARD FALL ____________

Scale:
0 point = Fails exercise 1a = Refuses, unsure or cannot lay down on the floor and rise independently or fails 1b.

1 point = Successful Exercise 1b = Can successfully lay down on the floor and rise independently but fails 2a or 2b

2 points =Successful Exercise 2b = Can successfully fall sideways from sitting down on the floor but fails 3a or 3b.

3 points = Successful Exercise 3b = Can successfully fall sideways from a squatting but fails 4a or 4b.

4 points = Can successfully fall sideways from a standing position without any harmful maneuver (exercise 4b).

"Strömqvist-Bååthe Test" for measuring Falling Competence Backwards, Sideways & Forward: Copyright: K. Strömqvist Bååthe & M. Tonkonogi, University of Dalarna, developed 2018 - 2019. English translation May 2023.
Tests may be used free of charge by trained test leaders with the acknowledgement of the correct test name as well as simple registration of the use by e-mail to: ksb@du.se stating: when and how the test will be used as well as name of point of contact, e-mail and name of institution/club/company

Name/Code: ________________ Date: ______ Testleader: ____________________ Place: _______________

Strömqvist-Bååthe Test - measuring Falling Competence (SBFC) – Forward:

1a) How confident are you laying down on your stomach and lift your head from the mat and put your arms beside you (palms down) and then stand up again?

☐ YES, SURE (go to 1b) ☐ UNSURE (stop test) ☐ NO/REFUSES (stop test)

1b) Performs the exercise – Lays down on the stomach, put the arms to the sides and lifts the head.

☐ Successful (go to 2a) ☐ Cannot get down on the stomach on the mat (stop test)
☐ Cannot lift the head off the mat (stop test)
☐ Cannot get up from the mat independently after laying down (stop test)

2a) How confident are you falling from a kneeling position or on all fours?

☐ YES, SURE (go to 2b) ☐ UNSURE (stop test) ☐ NO/REFUSES (stop test)

2b) Standing from a kneeling position or on all fours, no instructions given:

☐ Successful (go to 3a) ☐ Puts down hand/hands with straight arms (stop test)
☐ Does not hold up head (stop test) ☐ Falls on stomach (stop test)
☐ Hits the shoulder (stop test) ☐ Falls on elbows (stop test)
☐ Other injury prone maneuver: (describe) ____________________________________(stop test)

3a) How confident are you falling from a squatting position?

☐ YES, SURE (go to 3b) ☐ UNSURE (stop test) ☐ NO/REFUSES (stop test)

3b) Falling forward from a squatting position, no instruction given:

☐ Successful (go to 4a) ☐ Puts down hand/hands with straight arms (stop test)
☐ Does not hold up the head (stop test) ☐ Falls on the stomach (stop test)
☐ Hits the shoulder (stop test) ☐ Falls on elbows (stop test)
☐ Other injury prone maneuver: (describe) ____________________________________(stop test)

4a) How confident are you falling forward from standing up?

☐ YES, SURE (go to 4b) ☐ UNSURE (stop test) ☐ NO/REFUSES (stop test)

4b) Stands up and falls forward, no instruction given:

☐ Successful (=4p) ☐ Puts down hand/hands with straight arms (stop test)
☐ Does not hold up the head (stop test) ☐ Falls on the stomach (stop test)
☐ Hits the shoulder (stop test) ☐ Falls on elbows (stop test)
☐ Other injury prone maneuver: (describe) ____________________________________(stop test)

POINTS BACKWARD FALL ______________

Scale:
0 point = Fails exercise 1a = Refuses, unsure or cannot lay down on the floor and rise independently or fails 1b.

1 point = Successful Exercise 1b = Can successfully lay down on the floor and rise independently but fails 2a or 2b.

2 points =Successful Exercise 2b = Can successfully fall forwards from kneeling on the floor but fails 3a or 3b.

3 points = Successful Exercise 3b = Can successfully fall forwards from a squatting position but fails 3a or 4b.

4 points = Can successfully fall forward from a standing position without any harmful maneuver (exercise 4b).

"Strömqvist-Bååthe Test" for measuring Falling Competence Backwards, Sideways & Forward: Copyright: K. Strömqvist Bååthe & M. Tonkonogi, University of Dalarna, developed 2018 - 2019. English translation May 2023.
Tests may be used free of charge by trained test leaders with the acknowledgement of the correct test name as well as simple registration of the use by e-mail to: ksb@du.se stating: when and how the test will be used as well as name of point of contact, e-mail and name of institution/club/company
